# Recalibrating hope: A longitudinal study of the experiences of people with aphasia after stroke

**DOI:** 10.1111/scs.12745

**Published:** 2019-09-05

**Authors:** Felicity A.S. Bright, Clare M. McCann, Nicola M. Kayes

**Affiliations:** ^1^ Centre for Person Centred Research School of Clinical Sciences Auckland University of Technology Auckland New Zealand; ^2^ School of Psychology (Speech Science) University of Auckland Auckland New Zealand

**Keywords:** adjustment, aphasia, hope, stroke

## Abstract

**Background:**

Hope is a critical resource for people with aphasia after stroke, sustaining people though times of distress and uncertainty and providing motivation. In the first months after stroke, hope is vulnerable to different influences, and people can struggle to identify and work towards hopes for the future. We have little knowledge about how people with aphasia experience hope in the longer term after stroke.

**Objectives:**

To identify how people with aphasia experience hope 1 year after stroke and how hope may change in the year after stroke.

**Methods:**

The study used an Interpretive Description methodology. Interviews were conducted with four people with aphasia who had been interviewed 1 year previously. These were analysed using content analysis.

**Results:**

All people reported a broad sense of hope for the future. They described an active process of recalibrating their early poststroke hopes through a process of reflecting on past progress, current function and what they considered might be possible and desirable in the future. People were able to develop ‘new’ hopes that were meaningful and seemingly achievable when they had a sense of a possible, desirable future. Those who struggled to see a possible future maintained a hope that things will be good. Social supports, a sense of progress, engagement in meaningful activities and interactions appeared crucial in helping people (re)develop hopes for their future.

**Conclusions:**

Hope and hopes for the future gradually changed after stroke. Hope, identity and social connectedness were closely entwined and could enable people to both dwell in the present and move towards desired futures. This research suggests clinicians should prioritise creating hope‐fostering environments which support people to develop hope for their future.

## Introduction

Hope is a critical resource for people who have experienced a stroke 1, 2. Following a stroke, people commonly struggle to maintain or develop hope, perhaps reflecting the significant emotional consequences experienced by up to 70% of stroke survivors and the significant life changes that result 3, 4, 5, 6. Hope has been linked with improvements in emotional distress, motivation and engagement in rehabilitation 7, 8, treatment outcomes and quality of life 2, and is integral in ‘living successfully’ after a stroke 9, 10. Accordingly, it is an area deserving of closer consideration in research and clinical practice.

Hope can be understood in different ways, with multiple forms that commonly co‐exist. Research with people with aphasia after stroke suggested the presence of a broad sense of ‘simply having hope’ for the future, an inner state of hopefulness, was fundamental in sustaining them in the first weeks and months after a stroke 2, 7. Some people may also experience hope as ‘outcome oriented’ – a goal‐directed form of hope which pertains to particular desirable outcomes 2, 7. While people could hope for particular outcomes (for example, to return to work) in the postacute period after stroke, they did not consistently prioritise these nor expect them to be realised within the episode of rehabilitation. Rather, these outcome‐oriented hopes provided some direction for their efforts, giving them reason to engage in the rehabilitation process 7.

Few studies have explored how hope may change over time following a stroke 11. Certainly, it appears there may be differences over time 12. For instance, several studies have highlighted high levels of hopelessness in the early days following stroke 7, 11, 13. Some research suggests this is less evident later in recovery 7 although research with working‐age stroke survivors suggested people struggle to develop hope for the future, even years after their stroke 11. Instead, they maintained a sense of ‘realism’ to avoid future disappointment and ‘simply’ focused on living in the present, managing the impact of stroke 8, 11. Similar findings are evident in narratives of people 6 months after mild‐to‐moderate stroke 1. Having more comprehensive understandings of how and why people experience hope as they do may help clinicians critically consider their role in supporting hope, particularly in the later stages of stroke rehabilitation and recovery.

People with aphasia, who comprise up to 33% of people with stroke 14, may have unique experiences of hope after stroke. They have identified that hope is important for life after stroke 9, 10 and have suggested that relationships with others, being engaged in meaningful activities, and seeing progress can be key in fostering hope 7, 15, 16. However, the literature is clear that people with aphasia are more likely to be socially isolated after stroke 17, struggle to continue to participate in meaningful activities 18 and are more likely to have severe strokes, restricting possible impairment‐related recovery 19. All of these might influence their experience of hope. Hope may be particularly important in helping people to navigate these experiences, but at the same time it may be at its most vulnerable in this context. Hope is fostered through interactions with clinicians 7, yet is it well recognised that interactions are commonly impacted by aphasia, focusing on immediate needs rather than more existential issues 20, 21. For these reasons, it is critical to consider how this patient group experience hope over time.

Hope appears complex and changeable 3, challenging for patients and also for clinicians who describe tensions between supporting hope and wanting to foster realism 3, 22. Clinicians commonly value ‘hopes’ they consider are realistic and achievable, often within the short time period that they are working with the patient 22, 23. Stroke survivors commonly seek hope from their healthcare providers 7. However, because of the focus on realistic hope, providers can struggle to support people's broad hope 7, 22, 24 and can struggle to support them to (re)establish hope for their future 8. Developing a more comprehensive understanding of the experience of hope over time may enhance clinicians’ ability to have hope‐fostering interactions with their patients.

This study explores how hope was experienced by people with aphasia, 1 year after their stroke. It is part of a longitudinal study, which captured data in both the postacute and chronic phases of stroke. The findings of the postacute interviews have been reported elsewhere 7. The specific aims of the study were to identify: (1) how hope is experienced 1 year after stroke; and (2) how hope may change in the year after stroke.

## Methods

This study used an Interpretive Description methodology 25. Interpretive Description is a qualitative methodology used for small‐scale investigations of clinically relevant phenomena. Situated in a social constructionist epistemology, Interpretive Description ‘acknowledges the constructed and contextual nature of human experience that at the same time, allows for shared realities’ 25. It prompts researchers to identify patterns and commonalities that reflect the phenomenon, but also explain the variation that is likely present, producing descriptive findings that can inform clinical practice 26.

### Recruitment

All five participants in the previous study gave consent to be contacted for future‐related research and so were contacted by letter, inviting them to participate in this study. Four people responded, provided written consent and were interviewed.

### Data collection

Each participant was interviewed in their place of residence. The Western Aphasia Battery – Revised 27 was completed to identify the type and severity of any residual aphasia. Interviews were semi‐structured and lasted between 60 and 90 minutes. Questions explored the person's current experience of hope along with any perceived changes in hope since their previous interview. Questions included the following: ‘How do you feel about hope at the moment?’ and ‘How has your hope changed since we last talked?’. Prior to each interview, we reviewed transcripts of the initial interviews, allowing us to reorient participants to how they described hope at that time and to follow up on key ideas raised. For instance, one participant identified a hope of ‘being the best Dad he could be’. We asked whether this was still important, whether it was something he was actively working on and how this hope might have changed over the year. Supported communication strategies 28 facilitated communication. Interviews were audio‐recorded and transcribed. In the data presented in the Results, paraphasic errors (word and sound substitutions) are noted with the target (correct) word supplied in square brackets.

### Data analysis

Data were analysed using conventional content analysis 29 and constant comparison 25. Transcripts were read for familiarity. We initially analysed each participant's interview, looking closely for their experience of hope in the year after stroke. The transcripts were coded for keywords and ideas, and then, these codes were sorted into categories 29. To explore changes in hope after time, we then returned to the first interview with each participant, looking for similarities and differences between the two time points, guided by the participants’ own reflections on how hope had changed over time. Key similarities and differences were coded to capture the temporal element of hope. We then collated codes into categories. Through this process, the original categories were refined with some changes in the names and natures of some categories. Finally, by comparing and contrasting within and across categories, we created meaningful clusters which reflected the different patterns and commonalities within the data. Throughout analysis, we paid close attention to negative cases, people whose experiences of hope was markedly different to that of others and to people whose experiences of hope were markedly different between the two interviews (1 year apart). Through this, we constructed several themes that reflected participants experience of hope over time. The ongoing process of constant comparison within and between participants and within and between categories allowed us to identify patterns, commonalities and variations, as required in an Interpretive Description study 26. There was detailed discussion between the researchers throughout the analysis process to support our final interpretation.

### Rigour

Rigour was guided by Thorne's criteria of epistemological integrity, representative credibility, analytic knowledge and interpretive authority 25. This was sought through multiple means including: providing clear descriptions of the research process, prolonged engagement with the data, including listening to interviews throughout analysis, actively considering how the researcher was influencing data construction and analysis and incorporating raw data into text to demonstrate how analytic understandings were reached.

## Results

Four people with aphasia after stroke participated in this research. All were young stroke survivors, aged from 42 to 63 years at the time of this interview. All experienced residual stroke impairments. Their characteristics are presented in Table 1.

**Table 1:** Participant characteristics

	Gender	Age	Ethnicity	Type of stroke	Time poststroke	Residual symptoms	Aphasia severity and type	Living situation
Matthew	M	42	Pākehā (New Zealand European)	SAH, left MCA aneurysm	18 months	Aphasia Mild hemiparesis Cognitive changes – information processing, attention	AQ = 83 Anomic aphasia	Inpatient respite care
Adrienne	F	58	Pākehā	Left MCA infarct	18 months	Aphasia Mild hemiparesis	AQ = 80.2 Anomic aphasia	Living in own home with husband
Miriam	F	63	English	Left MCA infarct	17 months	Aphasia and apraxia of speech Dense hemiparesis	AQ = 57.3 Broca's aphasia	Living in own home with husband
Tony	M	48	Pākehā	Left MCA infarct, multiple small embolic infarcts	14 months	Nil evident	AQ = 94.8 Anomic aphasia	Living in own home alone

### Recalibrating hopes: an overview

All people reported a broad sense of hope that provided them with a sense of stability and possibility. This was present in both the postacute and chronic stages of recovery. They also reported a future‐oriented form of hope. They indicated an active process of recalibrating their hope for the future in the time since their stroke, revising hope (and hopes) in the light of their progress to date and their changing views and desires of their possible future. Accordingly, people were living in the present while both looking forward to the future and reflecting on their past. When people perceived progress to date and considered this was likely to continue *and* when they considered any loss resulting from the stroke was outweighed by their future possibilities, their hope was maintained, if not enhanced. Active and growing hopes for the future were evident in the narratives of those who were actively engaged in reconstructing their life, had a strong sense of identity and were developing (or had developed) a sense of their possible future.

### A broad sense of hope, providing stability throughout recovery

All four participants described hope as ‘essential’. Their sense of hope was long‐standing, persisting in the light of challenge. Miriam, who experienced the most severe aphasia and stroke‐related impairments, said ‘hope is the only way’. This broad sense of hope provided stability and grounding throughout recovery as people started to look to the future. In the face of adversity, their innate hope that things *could* be better appeared pivotal to getting through each day, suggesting it could have protective factors, supporting people to continue.

People's sense of hope was inherently entwined with their views of their past (specifically, their life and their recovery poststroke) and their present. Their sense of hope could change in response to this ongoing appraisal. While every participant reported a broad sense of hope at both interview points (at three and twelve months), this could be stronger or more vulnerable depending on how they perceived their recovery, and how they perceived what might be possible. For instance, people described an increasing hope for the future when they perceived they were making progress in ways meaningful to them. Matthew considered he had made significant progress in the year after his stroke: ‘Look at what I've achieved and I've achieved a lot, you know … it gives me more hope’. Similarly, Tony reported that his ‘continuation of improvement’ meant he could ‘dare to believe’ that his future would be positive even though aspects remained somewhat unknown and uncertain. However, when people perceived a lack of or inadequate progress, this could threaten their sense of hope. Miriam, who had experienced little improvement in her physical and language impairments, said ‘not enough progress has been to think about positive … it has to be positive to go forward … makes it much more difficult to be positive when you're [not improving] … [my hope is] a little bit backward’. While it was difficult to clarify Miriam's exact meaning, this quote suggested her sense of hope was reduced, as if it had ‘gone backward’ in the context of what she perceived as limited progress. Hope could also be threatened by unexpected medical events which could reinforce the tenuous nature of the future. Matthew described his hope after a series of seizures: ‘Oh my hope went down. I was just, I was just honestly, I thought, my God, I'm screwed, you know’. People's perceptions of their poststroke recovery, their well‐being, and view of the future, were crucial in fostering, or threatening, their sense of hope for the future.

### Active recalibration of hopes for the future: Identifying desired activities, roles and progress

Participants revisited what might be possible and desirable in the future based on their assessment of their past (specifically, their life and their recovery poststroke) and their present. Importantly, while the reference point for their comparison with the past was prestroke in the postacute phase, this shifted to the time immediately poststroke in the chronic phase. Their hopes were recalibrated in response to this ongoing appraisal. When reflecting on her experience of hope, Adrienne commented ‘hope is different now’, suggesting ‘there are new things to hope for’, even if these ‘things’ were not made clear. Specific hopes for the future were often similar to those identified in the early days after stroke, but took on different forms depending on their past experiences and views of the possible future. For example, at 3 months poststroke, Matthew wanted to be ‘the best dad I could be’. Nearly 1 year later, he had refined this, providing more information about what this might look like, such as having deeper conversations with his children and being able to relate well to them as they grow and change. Similarly, Miriam refined her initial broad hopes of ‘be the best I can be’ and ‘be less incapacitated’ to ‘positive results for (points to affected arm)’. Miriam's expression of hope appeared more guarded or limited when compared with other participants, perhaps reflecting that she had appraised her (limited) progress to date and had revised and confined her hopes in light of this. Indeed, later in the interview she described herself as ‘reluctant’ to hope based on her progress to date. This demonstrates how desired outcomes could be constrained, or extended, depending on how people perceived progress. What constitutes a desired outcome could also change relative to perceived progress. Matthew talked of returning to driving as a ‘ten hope’ but thought that when he got his licence back, that would ‘become an eight and there would be a new ten’ to aim for. It appeared that progress in areas meaningful to people was hope inspiring in and of itself and helped maintain and develop hope throughout recovery.

Three people considered their life had improved since their stroke. They appeared to believe more was possible and appeared to be actively hoping for ‘new possibilities’ as Adrienne described, hoping that life will continue to improve and that they will be able to engage in activities and roles meaningful to them. Common to these people was their sense of progress and the presence of social supports, both personal and professional. Hope and encouragement from others were essential for Matthew. He talked of actively evaluating people for their hope‐giving qualities, considering whether ‘they're the ones that are offering hope’, or if they should be in his ‘no hope basket’. Engagement with rehabilitation and stroke support services also appeared to expose them to others living active lives, which helped them both look for new possibilities and to ‘find’ a space where they belonged. Adrienne talked of her experience:

We have [service user] in our group and he hasn't spoken for 10 years … He doesn't [talk] … Cos not talking anymore, you have to reverse. You have to do something to … be a new person somehow … He hasn't listened [talked] for years and he's lovely and happy.

It appeared that seeing others living happily and meaningfully after stroke could open up a sense of the future being good and being something to look forward to and even model different ways of being. In contrast, Miriam had no social contact outside of her husband and child and rarely left the house. She had recalibrated her view of the future, from hoping for things to ‘be the best they can’ to being ‘survival focused’, focused on living ‘day by day’. These examples indicate how peoples’ hope and hopes did not sit in isolation; they were situated within and subject to the influences of their health and social context.

While people did reshape their early hopes (i.e. those identified in the first interviews at three months poststroke), it is important to note that these could co‐exist with new hopes, even when the person considered their early hopes were not going to be realised. This was particularly evident in Adrienne's narrative where she ‘hoped that speech would improve’, as well as having other hopes including ‘being a new person’. She considered her hope for improvement ‘does not make sense in my mind but it's true [that I still hope for improvement] … It's not right, it's wrong … it's wrong, how can I do that really, it's wrong’, as though self‐censoring her hopes. Over time, she had come to place less emphasis on the hope for improvement, but considered it important that hope could ‘still sit there at some level’, while she was also ‘getting on with life’. Forward‐oriented movement, not simply a return of prestroke function, appeared important, as was the ability to hold hope for improvement while not expecting or requiring these hopes to be realised.

A significant factor in *how* people recalibrated their hope was their corresponding journey of (re)construction of self and identity change, as indicated in Adrienne's comment: ‘I don't believe hope can be the same [over time] as you [are not] the same’. She considered the stroke gave her a chance to make changes to her life. She welcomed this, viewing it as an opportunity rather than an existential threat. People commonly reflected on their past, identifying they wanted to create a different life to their ‘old life’. Tony commented: ‘my hope is that I don't want to go back to it’ (his previous occupation). This was an uncertain time, but those with a strong sense of self appeared able to sit with uncertainty. For example, Tony expressed a sense of peace with himself and his circumstances: ‘I feel really at peace … I just feel very confident with who I am’. While uncertain, people hoped for ‘new possibilities’ as Adrienne described, gradually developing hopes related to the future they now considered possible, something distinct from life and function before and immediately after the stroke.

## Discussion

People with aphasia after stroke reported a continued process of recalibrating their hopes over time. This meant that they revisited their hopes for the future and adjusted these based on their sense of what was possible and desirable for the future, informed by factors such as their progress to date and their changing views of their possible future – a process of continued appraisal of the past, present and future which is not uncommon in stroke recovery (4, 30). In this chronic phase, when looking back to the past, their primary reference point was the time immediately poststroke and what had changed since then, with little attention to their life prestroke. This contrasts with their early poststroke narratives, where reflection back to their prestroke life and selves was dominant in their hopes for the future 7. The movement beyond life prestroke likely reflects their desire, and perhaps readiness and capacity, to move forward in life, actively constructing a future that is both meaningful and one they perceive as possible.

It is notable that people not only recalibrated their hopes, seemingly without prompting by clinicians, and were also able to hold both hopes for recovery and hopes for new possibilities with little issue and little expectation. This can challenge the literature which argues that acceptance and adjustment are important precursors for a person to move forward in their recovery 3, 31. Similarly, some clinicians seek to manage hope, commonly through a process of goal setting, to prompt patients to focus on what they consider realistic with the intention that this will help recovery and adjustment 22, 32. Our research suggests it is possible to both move on to explore future possibilities *and* hold on, not entirely giving up on things that are familiar and important. This reflects the ‘paradox of hope’ 11, 33, that a person may both acknowledge but also seek to defy some of the impacts of their condition. Indeed, defiance or resistance against outcome predictions has been shown to be important in adaptation in other neurological populations such as multiple sclerosis 34. It may be that focusing on what is ‘realistic’, often determined by what is perceived to be within a clinician's disciplinary scope of practice or possible to achieve in that particular episode of care 35, might not protect people from false hope in the way clinicians may think 3, 22 but instead limits people's ability to hold hope for the future. It has been suggested that when people feel their future outcome has already been determined, it can place them at risk of hopelessness as they see limited value in trying to change or improve things 34. We suggest that in trying to protect people's hope, there is a very real risk that clinicians could instead limit their hope and increase hopelessness and resignation about their future and stroke outcomes.

This research is clear: hope is social and relational in nature. The presence of social supports, relational connections and a sense of well‐being could mediate a lack of progress and appeared critical in supporting people to envisage future possibilities. This has significant implications for practice. First, it challenges clinicians to consider how their interactions and ways of being with people might either help to mediate or reinforce a sense of progress (or lack thereof). A focus on impairments (a common focus of rehabilitation 4), on what is *not* possible and what has been ‘lost’ might contribute to a sense of hopelessness if not accompanied by a sense of what might be possible and a focus on who and how a person can *be* with others. Second, it should prompt attention to social connections and relationships as mediating factors and how these are addressed within rehabilitation. For instance, social participation impacts not only on hope for the future, but also on quality of life and psychological well‐being 36, 37, 38 and can help provide continuity in the midst of discontinuity 4. However, this is an area not well addressed by rehabilitation services 4, 39. It is particularly problematic for people with aphasia, who experience reduced social networks and markedly worse social isolation compared with those without aphasia 17, 40. Rehabilitation services could consider what factors are important for helping people have hope and address these in treatment planning and by supporting patients to engage with different services and supports after formal rehabilitation ends. Clinicians might consider how they support people to maintain existing friendships, not just establish new connections through peer support groups, for instance 41. It is not to say that these are not important, and indeed, our participants reported a sense of hope from seeing others’ progress and from seeing others living happily even in the context of significant impairment. However, these findings support the call of previous researchers, that clinicians need to focus on supporting people to develop and maintain their social and relational networks 18, 38, 40, 42.

Hope appeared closely linked to people's sense of identity, and particularly, their reconstruction of self following their stroke. It is commonly recognised that people experience a sense of biographical disruption and loss of self after stroke 43, 44 even more so for those with aphasia 45. During rehabilitation, and indeed, in the years following, people are in a complex liminal space of being between old formulations of self, and the new and not‐yet‐known self 46. Reconstruction of self is aided by respecting personhood 43, social connectedness 43, 45, engaging in meaningful activities (including identifying *new* meaningful, valued activities and roles) 45, amidst other things. These are also the things that help people develop and maintain hope for the future, but which are also affected by aphasia 18. Developing cohesive narratives of the past, present and future helps people in identity reconstruction 43. It may be that holding the co‐existence of hopes for recovery and return of function with hopes for new possibilities described by our participants, helps provide a thread that enables people to maintain a sense of continuity and develop cohesive self‐narratives 43. Holding elements of the past, present and future may help people to have a sense of ‘dwelling’, ‘a form of being grounded in the present moment, supported by a past that is arising, and openness of a future that is calling’ (47, p. 548).

While we have shown relationships between hope, identity and social connectedness, and proposed different ways of working to support hope, we also suggest that different theories might help us make sense of the findings and provide a different framework to help clinicians consider hope in practice. ‘Dwelling‐mobility’^47^, ^48^ is a theory of well‐being based on Heidegger's work and proposes people experience the highest form of well‐being when they have a sense of both dwelling (in the present) and mobility (towards future possibilities). The ideas of dwelling and mobility resonate with our participants’ narratives of hope. They were evident in most of our participants who expressed a sense of contentment in their present situation, and while also holding a sense of future possibilities, an expectation they will be able to move forward in ways that are valued and meaningful 48. While most participants expressed a sense of ‘dwelling’, there was also a co‐existing ‘existential homelessness’ 47, 48, a loss of familiarity with the self which could either cause distress, as was evident in Miriam's interview, or provide an energising potential that prompts people to look towards the future with a sense of future mobility 47, 48. Hoping for future possibilities may be a critical component in how one navigates life after stroke in the context of existential threat to the self, providing a sense of mobility (or movement). Indeed, this theory might help us understand why people with stroke in Alazsewski and Wilkinson's 11 longitudinal hope study and Taule and colleagues’ 1 study with people with mild‐to‐moderate stroke experienced hope differently to participants in the current study; many appeared to be in a state of ‘existential homelessness’ with marked emotional support needs, without a co‐existing sense of dwelling or mobility. Their recalibration was based on what was considered no longer possible, rather than what might be possible or desirable. Attending to factors that help people ‘be’ in the present and look forward to future possibilities might help support people to have hope for the future and experience well‐being.

This paper is only the second to explore how people with aphasia experience hope 7 and the first to explore how hope changes over time in this population. This provides valuable insights that challenge clinicians to critically consider how they address hope in their clinical practice. This study is based on interviews with four participants. The findings therefore can be considered valuable but likely provide a partial understanding of how hope is experienced. All participants were Pākehā/New Zealand European and were working age. All had family support. It is likely that those in different situations and with different stroke outcomes might provide other perspectives. The study did not explore quality of life or mood; this might have provided useful information when interpreting the findings. Nevertheless, this research provides important information about hope, demonstrating its relationship to other aspects of recovery such as social participation and identity, and provides further evidence of its critical role in the years after stroke.

This study demonstrates that hope changes over time after stroke. It can take different forms: a broad sense of hope and an active hope for new possibilities in the future. Hoping for new possibilities appears particularly vulnerable to a sense of a lack of progress, social isolation and a loss of personal identity. The effects of aphasia may make hope even more vulnerable for those living with the effects of stroke. We suggest that directly addressing hope within rehabilitation planning and services is critical to help lay the foundations for people to have a sense of dwelling‐mobility, a sense of well‐being in the present and a sense of possible movement in the future.

## Author contributions

Felicity Bright: Designed the study, conducted data collection, led analysis, responsible for drafting the paper. Clare McCann: Designed the study, conducted data collection, contributed to analysis and drafting the paper. Nicola Kayes: Designed the study, contributed to analysis and drafting the paper.

## Ethical approval

Ethical approval for this research was obtained through an accredited Institutional Ethics Committee.
